# Efficient Prediction of Ki-67 Proliferation Index in Meningiomas on MRI: From Traditional Radiological Findings to a Machine Learning Approach

**DOI:** 10.3390/cancers14153637

**Published:** 2022-07-26

**Authors:** Yanjie Zhao, Jianfeng Xu, Boran Chen, Le Cao, Chaoyue Chen

**Affiliations:** 1 Department of Neurosurgery, West China Hospital, Sichuan University, No. 37, Guoxue Alley, Chengdu 610041, China; drzhaoyanjie@gmail.com (Y.Z.); cbrscu@foxmail.com (B.C.); 2 Department of Radiology, West China Hospital, Sichuan University, No. 37, Guoxue Alley, Chengdu 610041, China; 3 Department of Neurosurgery, Third People’s Hospital of Mianyang/Sichuan Mental Health Center, Mianyang 621054, China; xujianfeng3@sohu.com; 4 Department of Neurology, West China Hospital, Sichuan University, No. 37, Guoxue Alley, Chengdu 610041, China; lec445477@gmail.com

**Keywords:** Ki-67, magnetic resonance imaging (MRI), radiomics, machine learning, meningioma

## Abstract

**Simple Summary:**

A high Ki-67 index usually suggests accelerated and uncontrolled cell proliferation correlated with tumor growth and is a prognostic factor that is associated with an increased recurrent risk in meningioma patients. The aim of our study is to predict the Ki-67 proliferative index in meningioma patients using machine learning technology. With 371 cases collected from two centers, we systematically analyzed the relevance between clinical/radiological features and the Ki-67 index. Moreover, with radiomic features extracted from postcontrast images, we built three radiomic models and three clinical radiological–radiomic models to predict the Ki-67 status. The models showed good performance, with an AUC of 0.837 in the internal test and 0.700 in the external test. The results provide a quantitative method to facilitate clinical decision making for meningioma patients.

**Abstract:**

Background/aim This study aimed to explore the value of radiological and radiomic features retrieved from magnetic resonance imaging in the prediction of a Ki-67 proliferative index in meningioma patients using a machine learning model. Methods This multicenter, retrospective study included 371 patients collected from two centers. The Ki-67 expression was classified into low-expressed and high-expressed groups with a threshold of 5%. Clinical features and radiological features were collected and analyzed by using univariate and multivariate statistical analyses. Radiomic features were extracted from contrast-enhanced images, followed by three independent feature selections. Six predictive models were constructed with different combinations of features by using linear discriminant analysis (LDA) classifier. Results The multivariate analysis suggested that the presence of intratumoral necrosis (*p* = 0.032) and maximum diameter (*p* < 0.001) were independently correlated with a high Ki-67 status. The predictive models showed good performance with AUC of 0.837, accuracy of 0.810, sensitivity of 0.857, and specificity of 0.771 in the internal test and with AUC of 0.700, accuracy of 0.557, sensitivity of 0.314, and specificity of 0.885 in the external test. Conclusion The results of this study suggest that the predictive model can efficiently predict the Ki-67 index of meningioma patients to facilitate the therapeutic management.

## 1. Introduction

Meningioma is the most common type of intracranial tumor, which has an incidence rate of 37.6% among all primary central nervous system tumors [1,2,3]. According to the 2021 EANO guideline, surgery is considered to be the primary treatment of rapid growing meningioma, while observation is recommended for asymptomatic incidental tumors with a self-limited growth pattern [4]. The Ki-67 index, a histopathological marker defined by calculating the percentage of cells by immunostaining with a specific antibody in a section of confirmed tumor tissue, is reported to be significantly related to both treatment scheming and prognostic prediction [5,6,7]. A high Ki-67 index usually suggests accelerated and uncontrolled cell proliferation correlated with tumor growth, which is one of the main features indicating necessary clinical intervention [4,8]. Moreover, accumulated evidence has suggested that a high Ki-67 index is an independent prognostic predictor that is associated with an increased recurrent risk following surgical resection [9,10,11,12,13]. However, even though there is a correlation between the meningioma WHO grade and Ki-67 percentage, Ki-67 is not part of the WHO grading criteria of meningiomas. Therefore, an accurate prediction of the Ki-67 index status in meningiomas can facilitate clinical decision making and is important in individual treatment planning.

Magnetic resonance imaging (MRI) is the preferred modality for noninvasive detection and pretreatment diagnosis of meningiomas [4,14]. Previous research has shown that some MRI findings were useful in predicting the Ki-67 status for meningioma patients [15]. However, a manual analysis is subjective to both radiological variations and personal experience. A quantitative analysis with less interpretation by human expert evaluation is therefore warranted to better reflect intratumoral heterogeneity.

Radiomic analysis with machine learning has attracted considerable interest in neuro-oncological research [16]. Advances in the extraction of high-throughput computational features encourage oncologists to convert the gray-level intensity of clinical digital images into mineable data [17], which can be subsequently analyzed by machine learning algorithms [18,19]. Recently, evidence suggested machine learning was feasible to stratify the Ki-67 status in WHO grade I meningiomas based on a radiomic analysis from multiparametric MRI [20]. However, the following concerns remain to be addressed: first, the generalization of this method has not been tested on multicenter data; second, it remains unknown if the results are applicable to high-grade meningiomas; third, clinical and radiologic features were not yet incorporated into the model.

In this research, based on the standard preoperative MRIs collected from two institutions, we developed machine learning models to predict the Ki-67 proliferative index in meningioma patients. Moreover, clinical parameters and radiological findings were analyzed and introduced to the predictive models. The ability to predict Ki-67 preoperatively provides clinicians with fast yet important evidence that can be used to guide patient management and surgical strategy. 

## 2. Methods and Materials

### 2.1. Patient Selection

**Figure 1:**
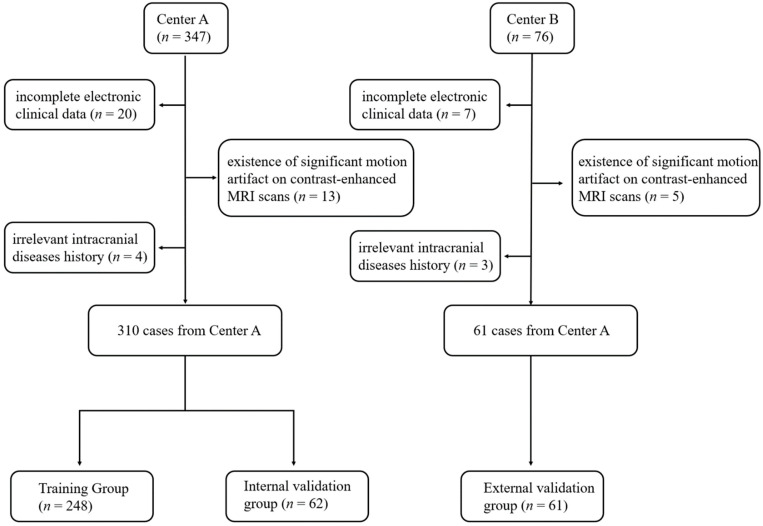
The flowchart of patient selection.

**Figure 2:**
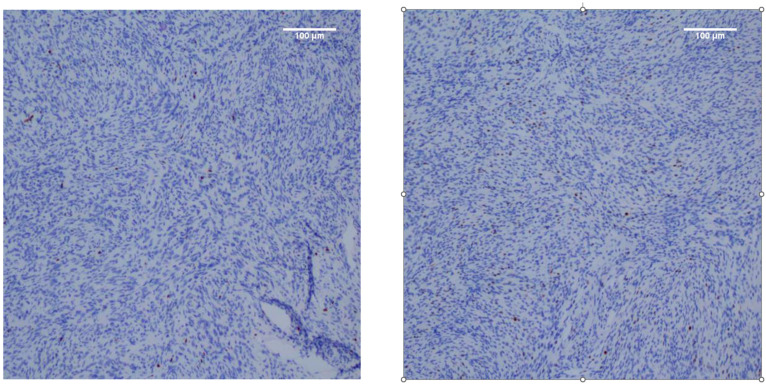
Immunohistochemical staining for Ki-67 in meningiomas.

This is a retrospective, multicenter study. From 1 January 2014 to 31 December 2020, 347 cases from Center A and 75 cases from Center B were initially collected in the present study. Their pathology reports were reviewed to ensure they met criteria for meningioma using the 2021 World Health Organization (WHO) Classification of Tumors of the Central Nervous System [2]. Inclusion criteria for selecting the subjects were as follows: (1) histologically confirmed meningioma and (2) available standard MR scans before any clinical intervention (including biopsy and radiotherapy). Exclusion criteria were (1) incomplete electronic clinical data (*n* = 27), (2) presence of significant motion artifact on MR scans (*n* = 18), and (3) irrelevant intracranial disease history, such as subarachnoid hemorrhage and cerebral infarction (*n* = 7). Based on the above criteria, 310 patients and 61 patients were identified from Center A and Center B, respectively. The flow chart of the patient selection process is demonstrated in Figure 1. In all patients, the Ki-67 labeling index was assessed by immunohistochemistry using an avidin–biotin–peroxidase complex method by using Aperio IHC image analysis software, as provided in Figure 2.

### 2.2. MR Scan Protocols

In Center A, standard MRI was performed in all patients on 3.0 T Siemens Trio Scanner. The detailed protocols and parameters were set as: Slice Thickness = 1 mm; Repetition Time = 1550; Echo Time = 1.98 s; Echo Number(s) = 1; Percent Phase Field of View = 90.625; Acquisition Matrix = 0\256\232\0; Flip Angle = 9 degrees.

In Center B, contrast-enhanced MRI was performed using 3.0 T Skyra. The detailed protocols and parameters were set as: Thickness = 1 mm; Repetition Time = 1550; Echo Time = 2.44 s; Echo Number(s) = 1; Percent Phase Field of View = 75; Acquisition Matrix = 0\256\154\0; Flip Angle = 8 degrees.

All the contrast-enhanced MR scans were acquired following the injection of gadopentetate dimeglumine (dose: 0.1 mmol/kg) as the contrast agent. The scanning of dynamic-enhanced MRI was conducted within 250 s after injection of the contrast agent. 

### 2.3. Image Preprocessing and Tumor Segmentation

Image preprocessing was required to standardize radiomic feature extraction. Image preprocessing for each of the patients included normalization at a scale of 100, a resampling of the images to 1 × 1 × 1 mm^3^ resolution, and gray-level intensity normalization in the range of 0 to 255. 

The study used 3D slicer software (version 4.11, Kikinis et al, Boston, MA, USA) to gain satisfying image segmentation. Among all MRIs, contrast-enhanced images can clearly describe the tumor boundary and were selected for radiomic feature extraction. Blinded to the electronic medical record and Ki-67 proliferation index, regions of interests (ROIs) were separately segmented along the boundary of the enhancing tumors by two neuroradiologists with more than 10 years of experience in image reading and were checked by a senior neuro-radiologist with more than 20 years of experience in image reading. Enhanced tumor dual tails were excluded in the ROIs in the present study.

### 2.4. Collection of Clinical Features, Radiological Features, and Radiomic Features

Five radiological features were analyzed by two neuroradiologists with more than 10 years of experience in image reading, including peritumoral edema, cerebrospinal fluid (CSF) space surrounding tumor, absent capsular enhancement, heterogeneous enhancement, and intratumoral necrosis. The following clinical features and pathological features were also retrieved: age, gender, and WHO grade. Tumor characteristics were calculated and collected from drawn ROIs, including laterality, location, maximum tumor diameter, and tumor volume.

The radiomic features were retrieved by using “PyRadiomics” package on Python. In total, 1218 radiomic features were initially retrieved, including shape features, first order radiomic features, and higher-order radiomic features from four different matrices, including gray-level cooccurrence matrix (GLCM), gray-level run length matrix (GLRLM), gray-level size zone matrix (GLSZM), and gray-level dependence matrix (GLDM). Then, the radiomic features were standardized by removing the mean and scaling to unit variance.

### 2.5. Feature Selection and Machine Learning Model Establishment

**Figure 3:**
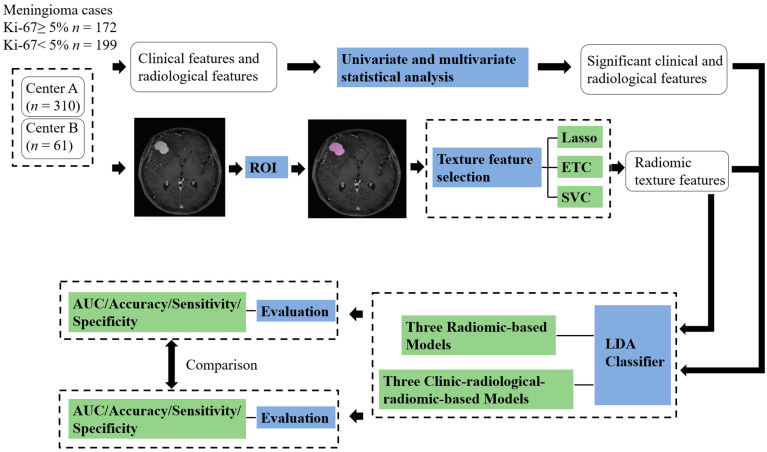
The workflow for the development of machine learning models to predict the Ki-67 index in meningioma patients. ETC: Extra tree classifier; Lasso: Least absolute shrinkage and selection operator; LDA: Linear discriminant analysis; SVC: Support vector machine; ROIs: Region of interests; AUC: Area under curve.

Figure 3 describes the workflow for establishing machine learning models. The extensive number of extracted texture features must be selected properly at first to avoid overfitting the machine learning algorithms. For clinical features and radiological features, multivariate logistic regression was performed to select the significantly correlated features for the machine learning model, and *p* values less than 0.05 were considered statistically significant in multivariate analysis. In addition, for radiomic features, three methods were independently used to select relatively important features, including least absolute shrinkage and selection operator (LASSO), extra tree classification (ETC), and linear support vector classification (LinearSVC).

Three radiomic-based machine learning models and three clinical radiological–radiomic-based machine learning models were established for five-fold cross-validation to predict the Ki-67 index in the meningioma patients. Cases from Center A were randomly divided into the training group and the internal test group at a ratio of 4:1; cases from Center B were used as the external test group. Although the Ki-67 index was determined as a prognostic predictor for meningioma patients, the optimal threshold had not been identified yet. Based on previous machine learning research, the Ki-67 index was stratified as binary variable by defining <5% as low and ≥5% as high [12]. The machine learning classifier used in this research was the linear discriminant analysis (LDA). Performances were demonstrated with areas under curve (AUC), accuracy, sensitivity, and specificity, respectively. The predictive models were performed with Python programming language (version 3.9).

### 2.6. Statistical Analysis

Categorical variables were presented with percentages and frequencies, whereas continuous variables were presented with means and standard deviation. In univariate analysis, point-biserial correlation analysis and chi-square test were used to assess the associations between the Ki-67 index and clinical/radiological features, and *p* value less than 0.10 were considered statistically significant. Interobserver agreement was evaluated by calculating intra-/interclass correlation coefficients (ICCs) of two extracted features, and only the radiomics features with high ICCs (ICCs ≥ 0.75) were taken into modeling. Statistical analysis was performed with IBM SPSS Statistics 22.

## 3. Results

### 3.1. Patient Characteristics

The patient baseline clinical characteristics and demographics are summarized in Table 1. The mean patient age was 52.6 ± 11.8 years (range: 5–82%), and the sex ratio of the study cohort was Male: Female =113: 258. For 310 cases from Center A, the mean Ki-67 of tumor specimens was 4.63 ± 2.96%, range between 1% and 20%. There were 152 patients (49.0%) with a low Ki-67 status and 158 patients with a high Ki-67 status (51.0%). For the 61 cases from Center B, the mean Ki-67 of tumor specimens was 3.57 ± 1.93% (range 1–10%), and a total of 14 subjects (23.0%) had a high Ki-67 status in this group. The vast majority of the included tumors were histologically proven as low-grade meningioma in both the high Ki-67 group (59.3%) and low Ki-67 group (87.9%).

### 3.2. Morphologic Analysis and Radiological Findings 

**Figure 4:**
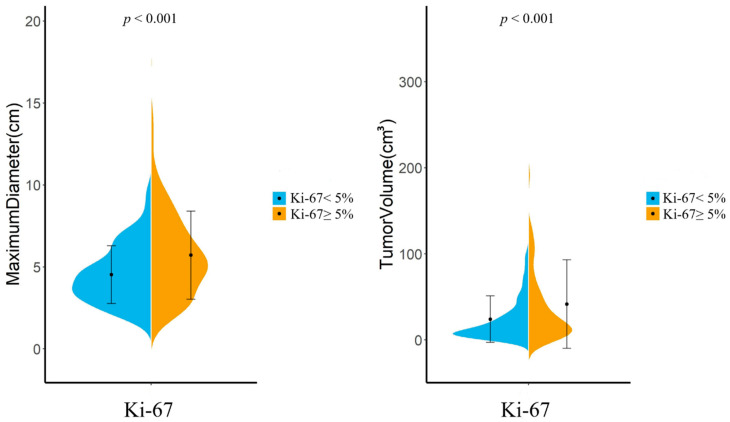
Distribution of maximum tumor diameter and tumor volume of Ki-67 ≥ 5% and Ki-67 < 5% meningiomas.

There were wide overlaps in both the maximum diameter and tumor volumes between lesions with a low Ki-67 index and a high Ki-67 index, as illustrated in Figure 4. The mean and standard deviation of maximum tumor diameters in high Ki-67 group were 5.72 ± 2.70 cm, compared with 4.53 ± 1.76 cm in the low Ki-67 group. Meningiomas with a high Ki-67 index were also larger in volume compared to tumors with a low Ki-67 index (41.57 ± 51.32 cm^3^ and 24.03 ± 27.00 cm^3^, respectively). Moreover, in the high Ki-67 group, the percentage of peritumoral edema, CSF space surrounding tumor, absent capsular enhancement, heterogeneous enhancement, and intratumoral necrosis was 78.5%, 58.1%, 25.0%, 59.3%, and 30.8%, respectively, while the percentages in the low Ki-67 group were 70.4%, 49.2%, 17.1%, 48.2%, and 22.6%, respectively.

### 3.3. Clinical and Radiological Features Related to Ki-67 Index

The results of the chi-square test and point-biserial correlation suggest that the presence of peritumoral edema (*p* = 0.076), CSF space surrounding tumor (*p* = 0.095), absent capsular enhancement (*p* = 0.072), intratumoral tumor necrosis (*p* = 0.078), heterogeneous enhancement (*p* = 0.037), higher WHO grade (*p* < 0.001), larger maximum tumor diameters (*p* < 0.001), and larger tumor volumes (*p* < 0.001) was significantly associated with a high Ki-67 status. A multivariate analysis of logistic regression suggested that intratumoral tumor necrosis (*p* = 0.032) and maximum tumor diameters (*p* < 0.001) were independently associated with the Ki-67 status. The results of the univariate analysis and multivariate analysis are demonstrated in Table 2.

### 3.4. Radiomic Feature Selection and Model Performances

Based on the results of feature selection, 14, 11, and 8 radiomic features were determined to be important and were separately introduced into predictive models wrapped by the LDA algorithm. The distribution of each selected feature is demonstrated in Table 3. Three radiomic-based models were constructed based on radiomic features, and three clinical radiological–radiomic-based models were constructed using different combinations of radiomic features and clinical features. The model performance in both the internal test and external test is listed in Table 4.

**Figure 5:**
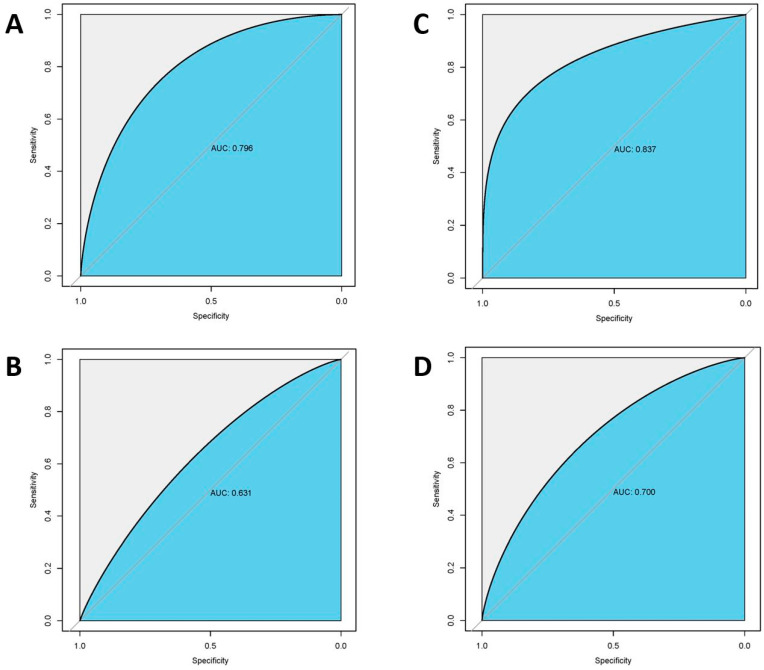
Receiver operating characteristic curves of Lasso+ LDA machine learning models. (**A**): Internal test (*n* = 62) of radiomic model; (**B**): External test (*n* = 61) of radiomic model; (**C**): Internal test (*n* = 62) of clinical radiological–radiomic-based model; (**D**): External test (*n* = 61) of clinical radiological–radiomic-based model; AUC: Area under Curve.

Among the radiomic-based models, a relatively better performance was yielded from the model constructed by the features selected by Lasso. In the internal test, the AUC, accuracy, sensitivity, and specificity were 0.795, 0.722, 0.724, and 0.719, respectively. In the external test, the model showed a decline in these indexes and the AUC of 0.631, accuracy of 0.508, sensitivity of 0.278, and specificity of 0.840. When combined with clinical features, this method showed improvement and achieved the highest performance among all the models, with AUC of 0.837, accuracy of 0.810, sensitivity of 0.857, and specificity of 0.771 in the internal test, and with AUC of 0.700, accuracy of 0.557, sensitivity of 0.314, and specificity of 0.885 in the external test. The ROC curves of the Lasso + LDA models are illustrated in Figure 5. All of the model performance is demonstrated in Table 4.

## 4. Discussion

Although the majority of meningiomas are classified as low-grade, benign tumors, there is wide heterogeneity in the rate of growth, clinical presentation, and risk of recurrence after treatment [21]. The prediction of Ki-67 is clinically relevant as it may reveal prognostic insights to predict tumor behavior and to assist in choosing a more individual treatment strategy [7,10,13]. In the current study, we systematically analyzed the relationship between the Ki-67 status and traditional radiological findings. Moreover, machine learning models fusing radiomic features and radiological features were trained to predict the Ki-67 status in meningiomas, and the performance of models was tested in both the internal cohorts and external cohorts. The results may guide surgical timing and operative strategy in that a more aggressive operative intervention with an earnest attempt should be considered for patients known to harbor tumors with a high Ki-67 status.

This study revealed five radiological features, which were significantly different between the high Ki-67 meningioma group and the low Ki-67 meningioma group. In our datasets, a univariate analysis suggested that compared to low Ki-67 meningiomas, high Ki-67 meningiomas were more likely to present peritumoral edema (*p* = 0.076), larger tumor volume (*p* < 0.001), and larger tumor maximum diameter (*p* < 0.001). These results are consistent with the long-held point that the rapid growth of tumors, for which a high-expressed Ki-67 is a surrogate marker, may induce a greater degree of peritumoral edema [15]. In addition, the results of this study also suggest that higher Ki-67 meningiomas were more likely to present intratumoral necrosis (*p* = 0.078) and heterogeneous enhancement (*p* = 0.037), which corroborated earlier findings that necrosis and “fluid-secreting” low-grade neoplasm were strong predictors of meningioma progression [22,23,24]. Another important finding was that absent capsular enhancement was significantly different between high Ki-67 and low Ki-67 meningiomas (*p* = 0.072), and this could result from the adaption of a rapid tumor, causing a less fibrous part and a more venous vascular component in the capsule [25]. Taken together, these morphologic findings indicated that tradition radiological features were useful in predicting Ki-67 status, highlighting the benefits of using clinical radiological–radiomic features instead of solely radiomic features to improve the performance of machine learning models.

There is a growing body of literature that has applied radiomic-based machine learning to meningiomas. These studies received promising results in prognostic analysis, grading prediction, and image-guided molecular diagnosis [5,6,7]. Generally, models involving multiparametric feature sets are superior to models involving single-sequence feature sets [26,27,28]. Similarly, in previous research, radiomic-based machine learning algorithms were built to predict the Ki-67 status in meningiomas by enrolling the features extracted from multiple MR sequences, including T1/T2-weighted, T1-weighted contrast-enhanced (T1CE), and FLAIR [20]. Their radiomic model outperformed our model with an AUC of 0.84. This result undoubtedly suggests that multiparametric feature sets could provide more information and assist in classification, which corroborated previous findings that some radiological features were more apparent on multiparametric MRI sequences. However, overfitting should be considered and investigated if the model can generalize the learning of the training data [20]. One major concern should be noted that there were too many features involved in their modeling compared to ours (60 vs. 14). The most convincing method to identify whether the trained model is overfitted is externally testing it on unseen data obtained from another institution [29,30]. The results of our external test suggest there was a moderate overfitting in our models, even if only 14 features were used as classifier inputs. Therefore, the generalization of the radiomic-based model was limited, and the improved method should be explored in future research. 

The present study further enrolled statistically significant clinical findings and radiological findings into the classifiers. The clinical radiological–radiomic-based models showed better performance in both the internal test and external test, with an AUC of 0.837 and 0.700, respectively. It has long been demonstrated in previous studies that some combined models may serve to outperform the single radiomic models or clinical models [31,32,33]. In contrast to the higher-order radiomic features, the relationship between these image parameters and tumor growth has long been established by researchers and provided a clearer interpretation of the models [25,34]. Considering that all the image findings included in this study can be easily collected in routine clinical practice, robust clinical radiological–radiomic-based models are more recommended to facilitate the treatment strategy and perform the surveillance of meningioma patients.

Our study has several limitations. First, this is a retrospective analysis, and inherent selection bias is inevitable. Second, the external test illustrates insufficiency in the sensitivity, as high Ki-67 patients only account for a small percentage of total patients in Center B. A large-scale validation from multicenter research is required to further support our results. Third, the methodology of this study is mainly restricted to machine learning algorithms, and advanced deep learning technology can provide an end-to-end approach without complicated preprocessing steps. Deep learning models should be investigated in future research. Finally, the model robustness should be examined in future studies. Since the radiomic features in the present study were extracted from MP-RAGE sequence, radiomics from different sequences including 3D-SPGR and TSE should be investigated.

## 5. Conclusions

This study set out to use machine learning algorithms to construct predictive models of the Ki-67 index before any invasive examinations in all grades of meningioma patients. We built three radiomic models along with three clinical radiological–radiomic-based models and proved them to be efficient and accurate. The findings will be of interest to the therapeutic management of meningioma patients in clinical practice. Further multicenter studies with advanced machine learning algorithms are required to validate the results.

## Figures and Tables

**Table 1:** Baseline clinical and radiological characteristics of the study population.

Characteristics	Center A (*n* = 310)		Center B (*n* = 61)		Total		*p* Value
	Ki-67 ≥ 5% (*n* = 158)	Ki-67 < 5% (*n* = 152)	Ki-67 ≥ 5% (*n* = 14)	Ki-67 < 5% (*n* = 47)	Ki-67 ≥ 5% (*n* = 172)	Ki-67 < 5% (*n* = 199)	
**Age**							
mean	51.7 ± 14.8	56.0 ± 10.0	51.7 ± 14.2	55.0 ± 12.1	51.7 ± 14.5	55.2 ± 11.7	0.546
range	5−82	39−76	9−77	31−77	5−82	31−77	
**Gender**							
male	53 (33.6%)	42 (27.6%)	5 (35.7%)	13 (27.7%)	58 (33.7%)	55 (27.6%)	0.215
Female	105 (66.4%)	110 (72.4%)	9 (64.3%)	34 (72.3%)	114 (66.3%)	144 (72.4%)	
**Location**							
Cerebral convexity	90 (57.0%)	78 (51.3%)	5 (35.7%)	25 (53.2%)	95 (55.2%)	103 (51.8%)	0.433
Falx	23 (14.5%)	32 (21.1%)	2 (14.3%)	7 (14.9%)	25 (14.5%)	39 (19.6%)	
Skull base	45 (28.5%)	42 (27.6%)	7 (50%)	15 (31.9%)	52 (30.3%)	57 (28.6%)	
**Laterality**							
Left	70 (44.3%)	69 (45.4%)	7 (50%)	21 (44.7%)	77 (44.8%)	90 (45.2%)	0.715
Right	71 (44.9%)	71 (46.7%)	6 (42.9%)	22 (46.8%)	77 (44.8%)	93 (46.7%)	
Midline	17 (10.8%)	12 (7.9%)	1 (7.1%)	4 (8.5%)	18 (10.4%)	16 (8.1%)	
**WHO grade**							
Low grade							
WHO I	94 (59.5%)	133 (87.5%)	8 (57.1%)	42 (89.4%)	102 (59.3%)	175 (87.9%)	<0.001
High grade							
WHO II	57 (36.1%)	19 (12.5%)	5 (35.7%)	5 (10.6%)	62 (36.0%)	24 (12.1%)	<0.001
WHO III	7 (4.4%)	0 (0%)	1 (7.2%)	0 (0%)	8 (4.7%)	0 (0%)	<0.001
**Peritumoral edema**	125 (79.1%)	110 (72.4%)	10 (71.4%)	30 (63.8%)	135 (78.5%)	140 (70.4%)	0.076
**CSF space surrounding tumor**	92 (58.2%)	78 (51.3%)	8 (57.1%)	20 (42.6%)	100 (58.1%)	98 (49.2%)	0.095
**Absent capsular enhancement**	39 (24.7%)	25 (16.4%)	4 (28.6%)	9 (19.1%)	43 (25.0%)	34 (17.1%)	0.072
**Heterogeneous enhancement**	93 (58.9%)	75 (49.3%)	9 (64.3%)	21 (44.7%)	102 (59.3%)	96 (48.2%)	0.037
**Intratumoral Necrosis**	48 (30.4%)	35 (23%)	5 (35.7%)	10 (21.3%)	53 (30.8%)	45 (22.6%)	0.078
**Maximum diameter**	5.76 ± 2.56	4.53 ± 1.63	5.16 ± 3.74	4.52 ± 2.13	5.72 ± 2.70	4.53 ± 1.76	<0.001
**Tumor volume**	43.1 ± 52.4	23.0 ± 26.5	24.8 ± 33.5	27.4 ± 28.6	41.57 ± 51.32	24.03 ± 27.00	<0.001

**Table 2:** Univariate and multivariate statistical analyses of clinical and radiological features.

Variables (Ki-67 ≥ 5% vs. Ki-67 < 5%)	Odds Ratio, 95% CI	*p* Value	
		Univariate Analysis	Multivariate Analysis
Peritumoral edema	1.538 (0.957–2.470)	0.076	0.279
CSF space surrounding tumor	1.403 (0.930–2.116)	0.095	0.216
Absent capsular enhancement	1.618 (0.976–2.681)	0.072	0.602
Heterogeneous enhancement	1.536 (1.035–2.361)	0.037	0.320
Intratumoral necrosis	1.524 (0.959–2.424)	0.078	***0.032***
Tumor volume (cm^3^)	1.013 (1.006–1.019)	<0.001	0.672
Maximum diameter (cm)	1.025 (1.014–1.035)	<0.001	***<0.001***

**Table 3:** The number of features selected by different feature selection methods.

Radiomic Features	Lasso (*n* = 14)	SVC (*n* = 11)	ETC (*n* = 8)
First-Order Features	3	3	0
Shape Features (2D)	1	1	0
Shape Features (3D)	0	0	0
GLCM Features	3	5	1
GLSZM Features	5	1	4
GLRLM Features	0	0	1
GLDM Features	2	1	2

GLCM: Gray-Level Co-occurrence Matrix; GLSZM: Gray-Level Size Zone Matrix; GLRLM: Gray-Level Run Length Matrix; GLDM: Gray-Level Dependence Matrix.

**Table 4:** Predictive model performance in the internal test and external test.

Features	Features	Test	AUC	Accuracy	Sensitivity	Specificity
Radiomics	Lasso + LDA	Internal Test	0.795 ± 0.033	0.722 ± 0.042	0.724 ± 0.043	0.719 ± 0.046
		External Test	0.631 ± 0.015	0.508 ± 0.027	0.278 ± 0.017	0.840 ± 0.019
	SVC + LDA	Internal Test	0.782 ± 0.034	0.730 ± 0.042	0.703 ± 0.058	0.769 ± 0.029
		External Test	0.646 ± 0.013	0.590 ± 0.021	0.323 ± 0.018	0.867 ± 0.030
	ETC + LDA	Internal Test	0.764 ± 0.038	0.645 ± 0.039	0.708 ± 0.033	0.605 ± 0.030
		External Test	0.56 ± 0.017	0.525 ± 0.032	0.143 ± 0.031	0.725 ± 0.23
Radiomics+ Clinics	***Lasso + LDA***	***Internal Test***	***0.837*** ± 0.036	***0.810*** ± 0.042	***0.857*** ± 0.040	***0.771*** ± 0.044
		***External Test***	***0.700*** ± 0.026	***0.557*** ± 0.027	***0.314*** ± 0.017	***0.885*** ± 0.030
	SVC + LDA	Internal Test	0.798 ± 0.033	0.698 ± 0.046	0.676 ± 0.056	0.731 ± 0.046
		External Test	0.702 ± 0.015	0.492 ± 0.017	0.282 ± 0.010	0.864 ± 0.014
	ETC + LDA	Internal Test	0.754 ± 0.024	0.710 ± 0.039	0.760 ± 0.038	0.676 ± 0.028
		External Test	0.607 ± 0.025	0.574 ± 0.027	0.286 ± 0.024	0.818 ± 0.021

ETC: Extra tree classifier; Lasso: Least absolute shrinkage and selection operator; LDA: Linear discriminant analysis; SVC: Support vector machine.

## Data Availability

Data are available on request due to privacy and ethical restrictions.
